# Work-related medical rehabilitation in patients with musculoskeletal disorders: the protocol of a propensity score matched effectiveness study (EVA-WMR, DRKS00009780)

**DOI:** 10.1186/s12889-016-3437-7

**Published:** 2016-08-17

**Authors:** Silke Neuderth, Betje Schwarz, Christian Gerlich, Michael Schuler, Miriam Markus, Matthias Bethge

**Affiliations:** 1 University of Applied Sciences Würzburg-Schweinfurt, Faculty of Applied Social Sciences, Münzstraße 12, Würzburg, 97070 Germany; 2 Institute of Social Medicine and Epidemiology, University of Lübeck, Ratzeburger Allee 160, Lübeck, 23562 Germany; 3 Department of Medical Psychology and Psychotherapy, Medical Sociology and Rehabilitation Sciences, University of Würzburg, Klinikstraße 3, Würzburg, 97070 Germany

**Keywords:** Work-related medical rehabilitation, Effectiveness, Work ability, Return to work, Propensity score matching

## Abstract

**Background:**

Musculoskeletal disorders are one of the most important causes of work disability. Various rehabilitation services and return-to-work programs have been developed in order to reduce sickness absence and increase sustainable return-to-work. As the effects of conventional medical rehabilitation programs on sickness absence duration were shown to be slight, work-related medical rehabilitation programs have been developed and tested. While such studies proved the efficacy of work-related medical rehabilitation compared with conventional medical rehabilitation in well-conducted randomized controlled trials, its effectiveness under real-life conditions has yet to be proved.

**Methods/Design:**

The cohort study will be performed under real-life conditions with two parallel groups. Participants will receive either a conventional or a work-related medical rehabilitation program. Propensity score matching will be used to identify controls that are comparable to treated work-related medical rehabilitation patients. Over a period of three months, about 18,000 insured patients with permission to undergo a musculoskeletal rehabilitation program will be contacted. Of these, 15,000 will receive a conventional and 3,000 a work-related medical rehabilitation. We expect a participation rate of 40 % at baseline. Patients will be aged 18 to 65 years and have chronic musculoskeletal disorders, usually back pain. The control group will receive a conventional medical rehabilitation program without any explicit focus on work, work ability and return to work in diagnostics and therapy. The intervention group will receive a work-related medical rehabilitation program that in addition to common rehabilitation treatments contains 11 to 25 h of work-related treatment modules. Follow-up data will be assessed three and ten months after patients’ discharge from the rehabilitation center. Additionally, department characteristics will be assessed and administrative data records used. The primary outcomes are sick leave duration, stable return to work and subjective work ability. Secondary outcomes cover several dimensions of health, functioning and coping strategies.

**Discussion:**

This study will determine the relative effectiveness of a complex, newly implemented work-related rehabilitation strategy for patients with musculoskeletal disorders.

**Trial registration:**

German Clinical Trials Register (DRKS00009780, February 10, 2016).

## Background

About 35 million people aged 15 to 64 (11.0 %) in the European Union are limited in the work they can do because of a long-standing health problem or a basic activity difficulty [1]. As musculoskeletal disorders are still one of the most important causes of work disability, various rehabilitation services and return-to-work programs have been developed and implemented in order to battle the consequences of musculoskeletal disorders for the opportunity to participate in working life [2]. As shown by systematic reviews, such programs and interventions indeed may significantly reduce sickness absence and increase sustainable return-to-work, especially if they include work-related elements [3–5].

In Germany, rehabilitation programs for musculoskeletal disorders are usually provided as three-week in- or outpatient interventions under the umbrella of the German Pension Insurance (GPI). The treatment is multi-professional and follows evidence-based therapy recommendations [6]. However, change in sickness absence duration was shown to be slight in meta-analyses of observational studies [7, 8]. One randomized controlled trial indicated no effect on sickness absence duration [9]. Moreover, several studies revealed that patients with severe restrictions of work ability (e.g. long-term sick leave, poor return-to work expectation) have a considerable risk of not returning to work despite participating in a conventional medical rehabilitation (CMR) program [10, 11]. Therefore, work-related medical rehabilitation (WMR) programs have been developed and tested in recent years [10, 12].

These programs were designed to particularly support patients with severe restrictions in terms of ability to work. WMR programs regularly comprise a diagnostic assessment that compares job demands and patients’ work capacity and offer therapeutic interventions such as work hardening and work-related functional capacity training, work-related psychosocial groups and intensified social counseling [13–16]. A meta-analytic synthesis of randomized controlled trials that compared WMR and CMR programs in Germany provides robust evidence that in patients with musculoskeletal disorders WMR programs achieve higher rates of sustainable return-to-work and reduce sickness absence after one year. WMR participants had 2.4 times higher odds of sustainable return-to-work after one year and also reduced sickness absence [17].

To support nationwide implementation of such programs and to improve rehabilitation outcomes, the GPI developed a WMR guideline. This guideline defines inclusion criteria for WMR (e.g. long-term sick leave, poor self-rated return-to-work expectation, unemployment) and important diagnostic and therapeutic measures in WMR (see Treatment section for a more detailed description of WMR) [13, 18]. The implementation of the WMR guideline was evaluated in patients with musculoskeletal disorders in seven rehabilitation centers. The results showed that the implementation was challenging but feasible. Moreover, the observational study showed a significant reduction of sick leave for WMR participants, and more work-related interventions predicted shorter sickness absence at the three-month follow-up [19]. However, findings also indicated that allocation and treatment decisions (WMR vs. CMR) only partly followed the guideline recommendations [20].

While studies proved the efficacy of WMR compared with CMR in well-conducted randomized controlled trials with high treatment fidelity and carefully selected patients, the effectiveness of WMR under real-life conditions has yet to be proved. Interventions that work in efficacy studies may not necessarily also do well in real-world applications [21, 22]. Though reliable efficacy studies are a necessary condition for evidence-based practice, patients and other stakeholders may be most interested in the effectiveness of real-world services. While a randomized controlled trial is the gold standard and the most robust way to prevent allocation bias in efficacy studies, other designs and methods may be needed to evaluate the effectiveness of an intervention, especially, as in the case of WMR, where nationwide dissemination is completed and programs and allocation procedures are established. Nevertheless, as the WMR guideline clearly describes the patients in need and proposes several screenings to identify these patients [11], allocation bias is a severe challenge in determining an unbiased treatment effect estimate. One proper evaluation design in this case is the use of observational data and a subsequent propensity score matched analysis to control for confounding. We therefore designed a cohort study to analyze the relative effectiveness of WMR compared with CMR. As current allocation procedures regarding WMR seem to be far from perfect and WMR and CMR patients have a considerable overlap, a propensity matched comparison was chosen to address allocation bias. We hypothesize that WMR reduces sickness absence and improves sustainable return-to-work and work ability ten months after rehabilitation (primary outcomes) compared with CMR. Moreover, we expect to see evidence of the superior effectiveness of WMR regarding several secondary outcomes. The study protocol has been prepared according to the SPIRIT checklist (Standard Protocol Items: Recommendations for Interventional Trials) [23].

## Methods

### Study design

The study is a cohort study under real-life conditions with two parallel groups. Participants will either receive a WMR or a CMR program in one of the 256 approved rehabilitation departments. The allocation ratio is determined by the actual allocation under real-life conditions of rehabilitation service provision in Germany. The current ratio of WMR to CMR patients is approximately one to five. The investigators will not have any influence on allocation decisions. Current utilization of WMR is still considerably below the estimated number of patients who need it. Therefore, comparable controls are available who receive a CMR, though their severe restrictions in terms of work ability would also justify participation in a WMR program. Although the researchers have no influence on the allocation procedure they will have the opportunity to model treatment allocation by observed data. Thus propensity score matching will be used to identify controls that are comparable to treated WMR patients and to estimate the unbiased effects of the relative effectiveness of WMR compared with CMR. As randomized controlled trials confirmed the relative efficacy of WMR in trials with high treatment fidelity and carefully selected patients, and implementation of WMR is completed, we assume the superiority of WMR.

Baseline data will be assessed after approval of the rehabilitation program but before the patients begin their rehabilitation. Follow-up data will be assessed three and ten months after their discharge from the rehabilitation center. Moreover, administrative data records will be used. Additionally, department characteristics will be assessed by a departmental survey. No one will be blinded before, during or after the trial.

### Study setting

All included rehabilitation centers are located in Germany. Rehabilitation services may be provided as inpatient or outpatient programs. Most of the departments are inpatient centers. There are some outpatient departments that are mainly located in major cities. Participation in a rehabilitation program was approved by the Federal GPI. In both groups, interventions will be performed by rehabilitation physicians, psychologists, physiotherapists, sports therapists, social workers, occupational therapists and other health professionals.

The duration of the rehabilitation program is initially determined by the Federal GPI (usually about three weeks). The rehabilitation center and the patient may arrange to extend the program. By request the patient may stop the rehabilitation program ahead of schedule.

### Treatment

#### Control

Participants of the control group will receive a CMR program according to current treatment standards and guidelines for the rehabilitation of musculoskeletal disorders. CMR programs last approximately three weeks. The daily quantum of therapy amounts to three or four hours. Following a multimodal approach, CMR programs include sports and exercise therapy, physiotherapy, occupational therapy, massage and other physical therapies, social and psychological counseling, patient education, pain management and relaxation training. CMR programs focus on the functional limitations of the musculoskeletal system and aim to restore physical abilities to promote participation in work and daily living. However, in contrast to WMR programs, they do not integrate an explicit focus on work, work ability and return to work in diagnostics and therapy.

#### Intervention

Participants of the intervention group will receive a WMR program according to the guideline for WMR [13, 18] as well as to the current treatment standards and guidelines for the rehabilitation of musculoskeletal disorders. WMR programs last approximately 2.4 days longer than CMR programs since they contain 11 to 25 h of work-related treatment modules [17]. Like CMR programs WMR programs follow a multimodal approach that comprises sports and exercise therapy, physiotherapy, occupational therapy, massage and other physical therapies, social and psychological counseling, patient education, pain management and relaxation training. However, WMR programs more explicitly focus on work, work ability and return to work. Thus, they include intensified work-related diagnostics as well as work-related functional capacity training, work-related psychosocial groups and intensified social counseling. Intensified work-related diagnostics identify individual needs by comparing work-related physical and psychosocial functional capacity with the patient’s job demands. Assessment of functional capacity is performed by means of a short functional capacity evaluation and questionnaires. Demands will be assessed by job analysis. Matching of capacity and demands is supported by standardized assessments [24–26]. Work-related functional capacity training exercises specific movements and postures according to individual workplace conditions. Work-related psychosocial groups deal with the mutual dependence of behavioral and emotional health experiences and the workplace environment. Additionally, preventative measures like stress management or conflict management in dealing with psychosocial stressors are taught. Finally, intensified social counseling examines the individual work-life situation and provides socio-legal guidance and advice concerning further assistance within the social security system.

### Participants

Patients are aged 18 to 65 years and have chronic musculoskeletal disorders, usually back pain. They have approval for rehabilitation either in an own rehabilitation institution of the Federal GPI or an institution that is leadingly occupied by the Federal GPI. Patients have requested rehabilitation because of health-related restrictions of work ability. Need for rehabilitation was acknowledged by a registered doctor and approved by the Federal GPI. Allocation to CMR and WMR is primarily decided by the socio-medical service of the Federal GPI. The socio-medical service may consider the findings of standardized screening which is usually part of the application documents and estimates the risk of not returning to work [11].

### Sample size estimation

**Fig. 1:**
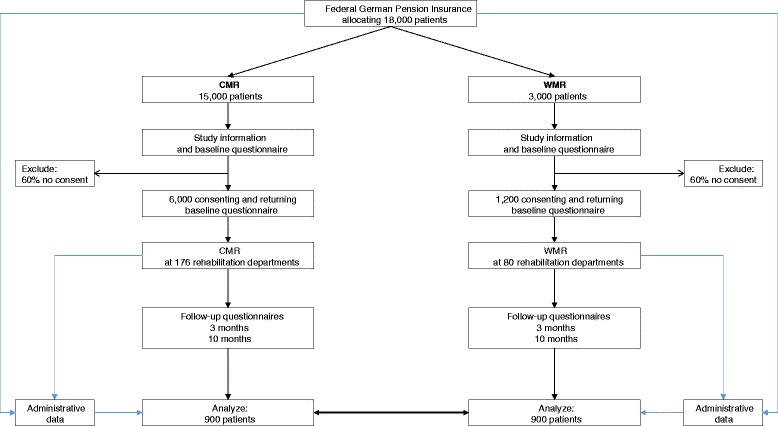
Flow of participants

We expect to find small mean differences between WMR and CMR in primary outcomes. For three months, of about 18,000 insured patients with permission for a musculoskeletal rehabilitation program 15,000 will receive CMR and 3,000 will receive WMR. We expect a participation rate of 40 %, i.e. 7,200 patients (6,000 CMR, 1,200 WMR) at baseline. At the 10-month follow-up, we expect a dropout rate of 25 %, i.e. 900 patients with WMR and 4,500 CMR left. Using a one-to-one match without replacement, 1,800 patients (900 WMR, 900 CMR) will be analyzed. Assuming a number of clusters (departments) *k* = 256 (with 176 departments providing CMR and 80 departments providing additionally WMR), a cluster size of *m* = 7.0, an intracluster correlation of rho = 0.05, a power of 0.80 and a Bonferroni corrected *p*-value = 0.016, an effect of SMD = 0.175 can be detected (Fig. 1).

### Recruitment

Participants with chronic musculoskeletal complaints whose request for rehabilitation was approved will be contacted by postal mail. Mails will be delivered in three waves at four-week intervals. All persons whose rehabilitation measure was approved within the previous four weeks will be contacted in each wave. The letter contains information about the study with the request to participate and the baseline questionnaire. The questionnaire contains no personal data but a unique study identifier. No personal data (e.g. name and address) will be submitted or published by the Federal GPI. One week after the initial postal contact a reminder will be sent to all potential participants. All participants will be thanked and reminded to participate if they have not yet done so. By this method, we expect to increase participant enrolment in order to reach our estimated target sample size and to strengthen external validity. Informed consent is assumed if patients complete their questionnaire and send the questionnaire to the research team. Additionally, participants will be asked to give permission to use administrative data from their pension insurance accounts and to link these data with the questionnaire data.

At the three- and ten-month follow-up participants who complete the baseline questionnaire will receive follow-up questionnaires from the Federal GPI (Table 1). Three weeks later the questionnaire will be sent again with a reminder to all participants who have not completed the questionnaire yet.

**Table 1:**
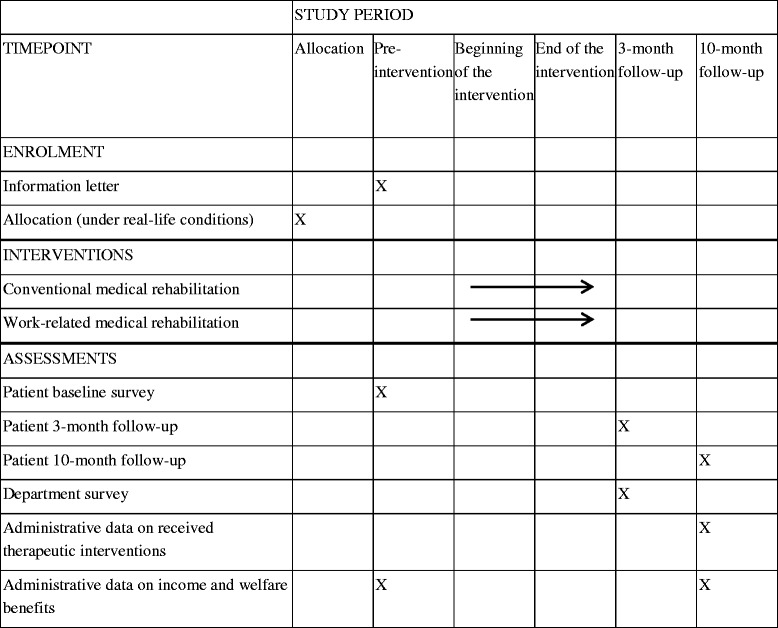
Schedule of enrolment, interventions, and assessments

### Data management

Questionnaires will be scanned and verified by an electronic data capture system and exported to statistical software packages for further analysis. Scanning and verifying will be done by trained research assistants. They will check electronically processed data item by item and compare imported data with the original questionnaire data. Administrative data will be extracted by the Federal GPI. Personal data will be removed and replaced by the unique study identifier. Data will be transferred to the principal investigator (MB). Finally, questionnaire and administrative data can be linked by the unique study identifier.

Data management will be done by the authors of the protocol. Data access is limited to the authors and the research assistants of the research team.

### Outcomes and other measures

This study will assess three primary outcomes as well as secondary outcomes, moderator variables and variables for propensity score matching. Outcomes and other measures will be assessed with patient questionnaires or will be extracted from administrative records (i.e. individual pension insurance accounts) provided by the Federal GPI. A complete list of all measured constructs, measurement points and expected scaling is shown in Table 2.

**Table 2:** Measures, assessment, expected scaling and measurement occasions

Outcome	Source and reference	Scaling	Baseline	3-month follow-up	10-month follow-up
Primary outcomes					
Sick leave duration in weeks	Own development	metric	X	X	X
Stable return to work	Own development	binary	X	X	X
Subjective work ability	WAS [28]	metric	X	X	X
Secondary outcomes					
General health	COPSOQ (1 item) [30, 31]	metric	X	X	X
Physical functioning	IRES-24 [32]	metric	X	X	X
Depression	PHQ-2 [33]	metric/binary	X	X	X
Anxiety	GAD-2 [34]	metric/binary	X	X	X
Pain intensity	CPQ [35, 36]	metric	X	X	X
Pain disability	CPQ [35, 36]	metric	X	X	X
Fear of movement	FABQ [37, 38]	metric	X	X	X
Physical activity	Modified Godin-Scale [39]	metric	X	X	X
Medication use	Own development	nominal	X	X	X
Self-management skills	heiQ [40]	metric	X	X	X
Employment	Own development	binary	X	X	X
Current sickness absence	Own development	binary	X	X	X
Subjective prognosis of employment status	SPE [41]	metric/binary	X	X	X
Implementation of work-related interventions	[19]	metric		X	
Consistency of the work-related strategy	[19]	metric		X	
Work-related benefit	[19]	metric		X	
Treatment satisfaction	CSQ-8 [42]	metric		X	
Income and welfare benefits	GPI accounts	metric	X		X
Therapeutic interventions	GPI accounts	metric			X
Department characteristics	Department survey	metric/nominal			X
Other measures					
Risk scores for not returning to work	SIMBO [11], WS [12]		X		
Somatization	SCL-90-R [43]	metric	X		
Pain generalization	Own development	metric	X		
Psychosocial stress	[44]	metric	X		
Socio-demographic data	Own development	metric/nominal	X		
Size of company	[45]	nominal	X		
Physical job demands	[46]	metric	X		
Effort-reward imbalance	[47]	metric	X		
Overcommitment	[47]	metric	X		
Support by co-workers and supervisors	Own development	metric	X		

Notes: *SCL-90-R* Symptom Check-List-90-R, *SIMBO* Screening-Instrument zur Feststellung des Bedarfs an medizinisch-beruflich orientierten Maßnahmen in der medizinischen Rehabilitation (Screening to assess the need for work-related medical rehabilitation), *COPSOQ* Copenhagen Psychosocial Questionnaire, *CPQ* Chronic Pain Grade Questionnaire, *CSQ*-8 Client Satisfaction Questionnaire, *FABQ* Fear-Avoidance Beliefs Questionnaire, *GAD*-2 Generalized Anxiety Disorder Questionnaire, *heiQ* Health Education Impact Questionnaire, *IRES*-24 Indikatoren des Reha-Status (Indicators for rehabilitation status), *PHQ*-2 Patient Health Questionnaire, *SPE* Subjective prognosis of employment status, *WAS* Work Ability Score, *WS* Würzburger Screening

#### Primary outcomes

The primary outcomes of this study are sick leave duration, stable return to work, and subjective work ability. Evaluation of the effectiveness of WMR relates to the 10-month follow-up. These three outcomes will also be assessed at the three-month follow-up and if appropriate at baseline.

To assess the duration of sick leave, the participants will be asked to report the number of weeks they have been off work for health reasons since discharge from the rehabilitation center. At baseline this question is related to the last 12 months. Stable return to work has been defined in accordance with Kuijer and colleagues [27] as a minimum of four weeks of employment without sick leave. Subjective work ability will be assessed by the Work Ability Score (WAS), which is the first item of the Work Ability Index (WAI) [28]. It compares current subjective work ability with the lifetime best. The 11-point scale ranges from zero (complete incapacity to work) to ten (lifetime’s best work ability). The WAS is highly correlated with the overall WAI score [29].

#### Secondary outcomes

Secondary outcomes cover several dimensions of health, functioning and coping strategies (see below) and will be measured at all three measurement points. At the three-month follow-up, participants will also evaluate how the rehabilitation programs dealt with work-related issues as well as satisfaction with the treatment.

##### General health

One item of the Copenhagen Psychosocial Questionnaire will be used to measure general health. The 11-point scale ranges from zero (worst imaginable health state) to 10 (best imaginable health state) [30, 31].

##### Physical functioning

Physical functioning is measured by eight items of the IRES-24 (German: Indikatoren des Reha-Status) [32], a widely used instrument in rehabilitation research in Germany. All items are measured on a five-point scale. Item values are averaged and rescaled to a range from zero to 10 points, with lower values indicating less functioning.

##### Depression and anxiety

The two-item versions of both the Patient Health Questionnaire (PHQ-2) and the Generalized Anxiety Disorder Questionnaire (GAD-2) will be used to assess depression [33] and anxiety [34]. The PHQ-2 uses the first two items of the PHQ-9 (“little interest or pleasure in doing things,” “feeling down, depressed or hopeless”) while the GAD-2 uses the first two items of the GAD-7 (“feeling nervous, anxious or on edge", “not being able to stop or control worrying”). All items are measured on a four-point scale (0 = not at all, 1 = several days, 2 = more than half of the days, 3 = nearly all days). Sum scores for depression and anxiety range from zero to six points. Additionally, we will determine binary outcomes by categorizing values of >2 as clinically relevant depressive or anxiety disorder.

##### Pain intensity and disability

Six items from the Graded Chronic Pain Scale will be used to assess pain intensity and pain disability [35, 36]. Pain intensity items cover current pain and worst pain in the last four weeks and average pain in the last four weeks. Pain disability is related to interference with daily activities, recreational, social and family activities and work activities. All items are scored from zero to 10 points, with higher scores indicating stronger pain or more disability. Items will be averaged and multiplied by 10. Thus, the composite scores range from zero to 100 points.

##### Fear of movement

Two items of the Fear-Avoidance Beliefs Questionnaire [37] (“My pain was caused by physical activity,” “I should not do physical activities which might make my pain worse”) will be used to assess fear of movement. Like Kent and colleagues [38], we will use scaling from zero (completely disagree) to 10 (completely agree) instead of the original scaling. A composite score is determined by averaging both item scores.

##### Physical activity

A modified version of the Godin Leisure-Time Exercise Questionnaire [39] will be used to assess how often per week and how long per session patients performed light, moderate and strenuous physical exercise. The total physical activity score in minutes per week will be calculated by multiplying the total number of sessions per week (in each domain) by the minutes per session (in each domain). Additionally, a total sum score will be calculated by multiplying the scores of each domain with the metabolic equivalent of tasks (9 = strenuous, 5 = moderate and 3 = light physical exercise).

##### Medication use

Medication used to reduce pain (e.g. Paracetamol), to enhance mood (e.g. Citalopram) or to treat other health complaints will be assessed by three new developed items. Response categories are no, regularly (e.g. daily) and rescue medication.

##### Self-management skills

Self-management skills will be assessed with three items of the scale *Skill and technique acquisition* of the Health Education Impact Questionnaire [40]. The items are measured on a four-point scale (1 = strongly disagree, 2 = disagree, 3 = agree, 4 = strongly agree). The scale score is the unweighted mean of all items, with higher values indicating higher subjective self-management skills.

##### Employment

To cover participation in working life the employment status (employed vs. unemployed) is asked for. Moreover, we will assess if the patients are on sick leave.

##### Subjective prognosis of employment status

Three items assess if patients believe they will remain at work until retirement, if patients assume that their health will be permanently jeopardized, and if patients intend to submit a request for a disability pension [41]. Items are summarized to reflect a total score ranging from zero to three points. Higher values indicate a more unfavorable outcome. A score of at least two points reflects that permanent work participation is deemed to be unlikely.

##### Perceived vocational orientation of the rehabilitation program

The realization of work-related goals and therapies during rehabilitation will be assessed with a slightly modified version of a previously used set of items from a study that investigated the implementation of the WMR guideline [19]. Participants report on 12 dichotomized items as to whether they received WMR contents throughout their rehabilitation program. Scores are aggregated to a total score ranging from zero to 12 points. This score reflects the implementation of the work-related therapies. Additionally, six items assess the perceived diagnostic and therapeutic focus on issues of return to work and work ability, e.g. the experience of a consistent return to work strategy. These items are measured on a five-point scale. Scores will be summed to a total score ranging from zero to 24 points. Finally, the subjective work-related benefit from participating in the rehabilitation program will be assessed by eight items measured on a five-point scale. Scores will be aggregated to a total score ranging from zero to 32 points.

##### Treatment satisfaction

Treatment satisfaction will be assessed using the German version of the Client Satisfaction Questionnaire [42]. This questionnaire includes eight items designed to assess various aspects of the patient’s satisfaction with the treatment. Items are measured on a four-point scale. The sum score ranges from eight to 32 points.

##### Income and welfare benefits

Income from regular employment and the duration of welfare benefits (for example, unemployment and sickness benefit) will be extracted from the GPI accounts.

#### Therapeutic interventions

Therapeutic interventions will be extracted from the standardized rehabilitation discharge letters [19]. These letters are stored in the individual GPI accounts. The documentation of the therapeutic interventions will indicate adherence to the WMR guideline.

#### Department characteristics

Measurement of department characteristics covers amongst others data on average guideline adherence and the number of treated patients. A survey of the departments will assess additional data on the department level (e.g. staff, team cooperation, and infrastructure).

#### Other measures

Additional measures will be assessed as potential effect modifiers and as variables for calculating the propensity score.

##### Risk of not returning to work

Two risk scores are frequently used to assess the need for WMR in Germany. The score of the SIMBO (German: Screening-Instrument zur Feststellung des Bedarfs an medizinisch-beruflich orientierten Maßnahmen in der medizinischen Rehabilitation) [11] ranges from zero to 100 points. Higher scores indicate a higher risk of not returning to work and a stronger need for WMR. A SIMBO score of ≥23 points was shown to be an optimal threshold to identify patients in need for WMR. The Würzburger Screening [12] states a risk of not returning to work and a need for WMR if a person is unemployed at the beginning of the rehabilitation program or scored one out of three points on a three-item scale that assesses negative return-to-work expectations.

##### Somatization

Somatization will be assessed by using seven items from the Symptom Check-List-90-R [43]. All items are measured on a five-point scale (0 = not at all, 1 = a little bit, 2 = moderately, 3 = quite a bit, 4 = extremely). Items are averaged to calculate a total score.

##### Pain generalization

Three newly developed items will assess the experience of widespread pain and pain amplification. These items are measured on a four-point scale (0 = totally disagree, 1 = disagree, 2 = agree, 3 = totally agree). Items are averaged to calculate a total score.

##### Psychosocial stress

Two items will assess family- and job-related stress in the last two weeks [44]. Both items are measured on a four-point scale (0 = not at all, 1 = several days, 2 = more than half of the days, 3 = nearly all days). Items are added to a sum score ranging from zero to six points.

##### Work environment

Several aspects of the work environment will be assessed as they might moderate the treatment effect: amongst others, size of company [45], physical job demands [46], effort-reward imbalance [47], overcommitment [47] and support by co-workers and supervisors.

##### Socio-demographic data

We will ask participants for socio-demographic data (age, sex, native language, educational level, partnership, children, and family life).

### Statistical analysis

#### Propensity score matching

Patients of WMR and CMR will be matched by propensity scores to achieve balanced sample characteristics and to calculate unbiased estimates of relative effectiveness [22, 48–54]. The propensity score is the conditional probability of receiving the treatment under evaluation (i.e. WMR) given the vector of observed background variables. Matching by propensity scores enables balanced characteristics of the treated and the untreated sample if there is sufficient overlap of the propensity scores of both groups. Compared with a conventional direct matching procedure the problem of multidimensionality in finding a corresponding control, for instance related to age, sex, sickness absence duration, pain, depression and others, is thereby reduced to one dimension only.

The propensity score will be estimated by a logistic regression model including all variables that are associated with the treatment allocation. For every WMR participant a patient with a similar propensity score will be selected from the larger pool of CMR patients. Resampling will be realized without replacement. If necessary to achieve balanced data, a caliper of one-quarter of the propensity score during resampling will be used. For sensitivity analysis, additional matching schemes will be tested (with and without replacement, different calipers, varied number of controls). As an indicator of the bias before and after matching owed to differences related to the observed sample characteristics the standardized percentage bias will be calculated. This is the difference of the sample means in cases and controls relative to the square root of the average of the sample variances in both groups [55]. Multiple imputations will be used to fill in missing data before estimation of the propensity score. The propensity scores for each record will be averaged across the completed datasets, and propensity score matching will be performed with these averaged scores [56].

#### Multilevel regression analyses

Analyses of treatment effects in propensity score matched samples can use the same statistical methods as those used in experimental studies [48, 49]. Multilevel regression analyses will be used to account for dependencies in the data [57, 58]. Individual patient data will be conceptualized as level-1 parameters and the rehabilitation department as a level-2 variable. All models will include the treatment variable (WMR vs. CMR), the baseline score of the respective outcome measure and a random intercept which reflects the rehabilitation department. For the primary outcomes, p-value will be fixed at 0.016 (Bonferroni corrected for three outcomes). For the secondary outcomes, p-value will be fixed at 0.05. Linear models will be used for continuous outcomes and logistic models for binary outcomes.

#### Moderator analyses

Exploratory moderator analyses will be performed to clarify whether level-1 or level-2 characteristics modify the treatment effect. The modeling of moderator effects of level-1 characteristics will be realized by multiplicative interaction terms of the treatment indicator and potential moderators. Moderator effects of level-2 variables will be tested by including the potential moderator as a level-2 predictor of the level-1 treatment-effect. In the case of continuous moderators, these variables will be z-standardized [59]. Level-1 moderators will be tested in order to identify patients who particularly benefit from WMR. Level-2 moderators may suggest department characteristics that may impact the effectiveness of WMR.

## Discussion

This study will provide an estimation of the relative effectiveness of a complex, newly implemented work-related rehabilitation strategy for patients with musculoskeletal disorders. Findings will complement the existing evidence of the relative efficacy derived from randomized controlled trials by robust estimation of the effects under real-life conditions of rehabilitation service provision in Germany. The findings of this study will be published in peer-reviewed journal articles and conference presentations.

## Trial status

Recruitment has started and is ongoing.

## Abbreviations

CMR, conventional medical rehabilitation; GAD-2, Generalized Anxiety Disorder Questionnaire; GPI, German Pension Insurance; IRES-24, Indikatoren des Reha-Status; PHQ-2, Patient Health Questionnaire; WAI, Work Ability Index; WAS, Work Ability Score; WMR, work-related medical rehabilitation
